# Relationship between fear of COVID-19 and individual factors – a preliminary study

**DOI:** 10.1192/j.eurpsy.2021.694

**Published:** 2021-08-13

**Authors:** C. Cabaços, A.T. Pereira, A. Araujo, R. Sousa, A. Macedo

**Affiliations:** 1 Psychiatry Department, Centro Hospitalar e Universitário de Coimbra, Coimbra, Portugal; 2 Institute Of Psychological Medicine, Faculty Of Medicine, University of Coimbra, coimbra, Portugal; 3 Usf Coimbra Centro, USF Coimbra Centro, Coimbra, Portugal

**Keywords:** COVID-19, Fear of COVID-19, Personality, Repetitive negative thinking

## Abstract

**Introduction:**

Fear associated to infectious diseases is directly related with their transmission rate, morbidity and mortality. High levels of fear associated with COVID-19 can affect people’s ability to act and think rationally. In a time of pandemics, it is essential to understand individual factors that might be associated to higher vulnerability to stress and fear.

**Objectives:**

To analyse: a)correlations between Fear of Covid-19 and clinical and sociodemographic characteristics; b)the mediator role of repetitive negative thinking on the relationship between personality traits and Fear of Covid-19.

**Methods:**

234 adults (75.6% women; mean age=29.53±12.51) completed an on-line survey with the Portuguese version of the Fear of Covid-19 Scale (FCV-19S) and other questionnaires to evaluate clinical and sociodemographic characteristics (years of education, perceived physical and mental health and infection by Covid-19), Personality (NEO-FFI-20) and Repetitive negative thinking (PTQ-15). SPSS and Process Macro (Hays, 2020) were used.

**Results:**

FCV-19 mean scores were significantly higher in women and significantly correlated with years of education (r=-.14) (p<.05). History of previous/current Covid-19 infection did not significantly distinguish FCV-19 scores and they did not correlate with perceived health. FCV-19 correlated significantly with neuroticism and PTQ total and dimensional scores (r>.20, p<.01). Both Repetitive thinking and Cognitive interference were mediators of the relationship between neuroticism and fear of COVID, even after controlling for gender and education.

**Conclusions:**

This study provides preliminary evidence on individual factors that might be associated to the emotional response to the Covid-19 pandemics, aiming to facilitate public health initiatives to ease people’s fears in a near future.

